# VEGF-A enhances the cytotoxic function of CD4^+^ cytotoxic T cells via the VEGF-receptor 1/VEGF-receptor 2/AKT/mTOR pathway

**DOI:** 10.1186/s12967-023-03926-w

**Published:** 2023-02-03

**Authors:** Ziyi Chen, Meng Zhang, Yufeng Liu, Zhe Chen, Ling Wang, Wenjuan Wang, Jincheng Wang, Mingqian He, Bingyin Shi, Yue Wang

**Affiliations:** 1 Department of Endocrinology, The First Affiliated Hospital of Xi’an Jiaotong University, Xi’an, China; 2 MOE Key Lab for Intelligent Networks & Networks Security, School of Electronic and Information Engineering, Xi’an Jiaotong University, Xi’an, China; 3 Genome Institute, The First Affiliated Hospital of Xi’an Jiaotong University, Xi’an, China; 4 BioBank, The First Affiliated Hospital of Xi’an Jiaotong University, Xi’an, China; 5 Department of Spine Surgery, Hong Hui Hospital, Xi’an Jiaotong University, Xi’an, China; 6 Department of Hematology, The First Affiliated Hospital of Xi’an Jiaotong University, Xi’an, China

**Keywords:** CD4^+^ cytotoxic T cells, CD4 CTLs, VEGF-A, VEGF-R1, VEGF-R2, mTOR, Graves orbitopathy

## Abstract

**Background:**

CD4^+^ cytotoxic T cells (CD4 CTLs) are CD4^+^ T cells with major histocompatibility complex-II-restricted cytotoxic function. Under pathologic conditions, CD4 CTLs hasten the development of autoimmune disease or viral infection by enhancing cytotoxicity. However, the regulators of the cytotoxicity of CD4 CTLs are not fully understood.

**Methods:**

To explore the potential regulators of the cytotoxicity of CD4 CTLs, bulk RNA and single-cell RNA sequencing (scRNA-seq), enzyme-linked immunosorbent assay, flow cytometry, quantitative PCR, and in-vitro stimulation and inhibition assays were performed.

**Results:**

In this study, we found that VEGF-A promoted the cytotoxicity of CD4 CTLs through scRNA-seq and flow cytometry. Regarding the specific VEGF receptor (R) involved, VEGF-R1/R2 signaling was activated in CD4 CTLs with increased cytotoxicity, and the VEGF-A effects were inhibited when anti-VEGF-R1/R2 neutralizing antibodies were applied. Mechanistically, VEGF-A treatment activated the AKT/mTOR pathway in CD4 CTLs, and the increases of cytotoxic molecules induced by VEGF-A were significantly reduced when the AKT/mTOR pathway was inhibited.

**Conclusion:**

In conclusion, VEGF-A enhances the cytotoxicity of CD4 CTLs through the VEGF-R1/VEGF-R2/AKT/mTOR pathway, providing insights for the development of novel treatments for disorders associated with CD4 CTLs.

**Supplementary Information:**

The online version contains supplementary material available at 10.1186/s12967-023-03926-w.

## Introduction

CD4^+^ cytotoxic T cells (CD4 CTLs) are CD4^+^ T cells with major histocompatibility complex (MHC)-II-restricted cytotoxic function possessing a variety of surface markers. Since their discovery in vitro in 1981 and ex vivo in 2004, CD4 CTLs have been reported to be present in both healthy and pathologic conditions [1, 2]. Taniuhi et al. discovered that healthy individuals of all ages (including supercentenarians) had CD4 CTLs in their peripheral blood mononuclear cells (PBMCs) [3]. Additionally, autoimmune diseases, malignancies, and acute and chronic viral infections make up the disorders in which CD4 CTLs play roles [2, 4, 5]. By killing target cells, CD4 CTLs under pathologic conditions, which exhibit upregulated cytotoxic and proinflammatory molecules (e.g., granzyme, perforin, interferon-γ, etc.), are able to control tumor growth and viral infection as well as hasten the progression of autoimmune diseases [6, 7]. Therefore, to better understand the pathogenesis of various diseases and develop novel treatments, it is crucial to investigate the effects and regulatory mechanisms of CD4 CTLs.

Recently, an increasing number of researchers have paid attention to the regulators of the cytotoxicity of CD4 CTLs and revealed that the T cell receptor (TCR), costimulatory/inhibitory molecules and cytokines are potential mediators. Constant exposure to antigens has been shown to enhance the levels of cytotoxic molecules in CD4^+^ T cells, accompanied by loss of T-helper-inducing POZ/Krueppel-like factor (ThPok, a crucial transcription factor for phenotype maintenance in CD4^+^ Th cells), which implied a potential role for TCR signaling in the cytotoxicity of CD4 CTLs [8]. Moreover, when agonists specific for the costimulatory molecules CD134 and CD137 were applied in mouse tumor models, eomesodermin (Eomes), which promotes the production of granzyme (Grm)B, was upregulated in CD4 CTLs [9]. Regarding cytokines, IL-15, IL-2, interferon (IFN)-I and IL-18 may enhance the cytotoxic function of CD4 CTLs via the Janus kinase (JAK)/signal transducers and activators of transcription (STAT) pathway [10–12]. However, even after numerous studies have been carried out, the regulators of the cytotoxic function of CD4 CTLs have still not been completely elucidated.

In a previous study, we discovered that CD4 CTLs had elevated cytotoxic function in patients with Graves orbitopathy (GO), indicating that there might be regulators of the cytotoxicity of CD4 CTLs in GO [13]. Here, by analyzing RNA-sequencing (RNA-seq) data for PBMCs from patients with GO or Graves hyperthyroidism (GH), we identified the vascular endothelial growth factor (VEGF) A gene as a regulator of the cytotoxic function of CD4 CTLs in GO. Furthermore, single-cell RNA sequencing (scRNA-seq) and in vitro VEGF-A stimulation assay data analysis demonstrated that VEGF-A enhanced the cytotoxicity of pan CD4 CTLs. Mechanistically, we discovered that VEGF-A, via the VEGF-receptor (R)1/2 and AKT/mTOR pathway, enhanced the cytotoxic activity of CD4 CTLs. These findings suggest that VEGF-A contributes to the enhancement of the cytotoxic function of CD4 CTLs and may serve as a therapeutic target in disorders involving CD4 CTLs.

## Material and methods

### Study subjects

Twenty-seven treatment-naïve GO patients, 24 treatment-naïve GH patients, and 14 healthy controls (HCs) were enrolled in this study. Regarding those recruited samples from GO patients, 20 were used for RNA-sequencing, 4 were for in-vitro VEGF-A stimulation assays, 3 were for ELISA assays, 7 were for VEGF-R-related assays, and 5 were for phosphorylated molecules-related assays. The GO and GH patients were recruited from the Department of Endocrinology, The First Affiliated Hospital of Xi’an Jiaotong University. This study was approved by the Ethics Committee of the First Affiliated Hospital of Xi’an Jiaotong University. We obtained informed consent from each patient and HC after explaining the purpose of our study.

### Primary CD4^+^ T-cell isolation and culture

For primary CD4^+^ T-cell isolation, PBMCs were acquired utilizing Ficoll Paque Plus (GE Healthcare, USA) gradient cell separation according to the manufacturer’s instructions. Next, the CD4^+^ T Cell Isolation Kit, human (Miltenyi Biotec, Germany) was utilized to isolate CD4^+^ T cells. Then, the cells were resuspended at 1 × 10^6^ cells/mL and cultured in advanced RPMI1640 medium (Gibco, USA) supplemented with 10% fetal bovine serum (FBS; Gibco), 1% 2-mercaptoethanol (2-ME; Gibco) and 1% penicillin–streptomycin (Gibco) in 48-well plates precoated with 2 μg/mL anti-human CD3 antibody (BioLegend, USA). For VEGF-R-related assays, after incubation with 5 μg/mL anti-VEGF-R1 antibody (R&D Systems, USA) and/or 10 μg/mL anti-VEGF-R2 antibody (Abcam, USA) for 1 h, CD4^+^ T cells were treated with blank (CON) or 15 ng/mL VEGF-A (R&D Systems, USA) for 2 days respectively. GolgiPlug Protein Transport Inhibitor (BD Bioscience, USA) was administered for the last 6 h to detect the cytotoxic molecules. Regarding AKT/mTOR inhibition assays, CD4^+^ T cells were treated with blank (CON), 15 ng/mL VEGF-A, VEGF-A + 10 μM MK-2206 2HCl (AKT inhibitor, MedChemExpress, USA), VEGF-A + 100 nM rapamycin (mTOR inhibitor, Selleck, China) or VEGF-A + 10 μM MK-2206 2HCl + 10 μM MHY1485 (mTOR activator, MedChemExpress, USA) for 2 days in the presence of GolgiPlug Protein Transport Inhibitor for the last 6 h.

### Flow cytometry

Cells were resuspended at a final concentration of 1 × 10^7^ cells/mL in flow cytometry staining buffer (eBioscience, USA). After blocking with TruStain FcX PLUS (BioLegend), surface staining was performed at 4 °C for 30 min. For intracellular staining, a transcription factor buffer set (BD Bioscience) was used to fix and permeabilize cells according to the manufacturer’s instructions, and the cells were then stained for intracellular molecules at 4 °C for 45 min. Stained cells were run on a Canto II (BD Bioscience) and analyzed using FlowJo software version 10.7.2 (TreeStar).

For phosphorylated protein analysis, cells were fixed with 4% cold PFA for 10 min at room temperature, permeabilized and blocked with PBS buffer containing 0.1% Triton X-100 and 2% BSA for 1 h at 4 °C. Next, the cells were stained with primary antibodies for 1.5 h at 4 °C, followed by incubation with secondary antibodies and antibodies targeting cell-surface markers for 30 min at 4 °C. PBS buffer containing 0.1% Triton X-100 and 0.5% BSA was utilized to wash the cells.

The following monoclonal antibodies were used: anti-human CD4, anti-killer cell lectin-like G1 (KLRG1), anti-human granzyme GrmB, anti-human GrmA, anti-human perforin (Prf), and anti-human neuropilin (NRP)-1 from BioLegend; polyclonal anti-human VEGF-R1 from R&D Systems; polyclonal anti-human VEGF-R2 and an anti-rabbit IgG (H+L) secondary antibody from Abcam; anti-phosphorylated (p)-AKT from Cell Signaling Technology; anti-p-mTOR and anti-p-S6 kinase (S6K) from eBioscience; and anti-goat IgG (H+L) from Proteintech.

### Enzyme-linked immunosorbent assay (ELISA)

Plasma was collected from GO patients, GH patients, and HCs for ELISA analysis to quantify the concentration of VEGF-A. After dilution, plasma samples were added to an ELISA plate precoated with 2 μg/mL anti-VEGF capture antibody (R&D Systems) and incubated for 4 h at room temperature. Next, 0.25 μg/mL biotinylated anti-VEGF-165 (the most common isotype of VEGF-A in humans) affinity-purified antibody (R&D Systems) was added and incubated for 1 h at room temperature [14]. After incubation with an Av-HRP conjugate, TMB reagent was added, and the optical density (O.D.) for each well was read with a microplate reader (KHB, China) set to 450 nm.

### Quantitative PCR (qPCR)

A MACS bead system (Miltenyi) was used to sort CD4^+^KLRG1^+^ and naïve CD4^+^ T cells according to the manufacturer’s guidelines. Sorted cells were confirmed to be > 85–95% pure. RNA used for reverse transcription was extracted from approximately 100,000–500,000 sorted CD4^+^KLRG1^+^ from GO patients and CON/MHY1485 (an mTOR activator)/VEGF-A-stimulated samples or HC naïve CD4+T cells using the Direct-zol RNA Microprep Kit (Zymo Research, USA). The Prime Script Master Mix Kit (Takara, Japan) was used to synthesize cDNA according to the manufacturer’s protocol, followed by qPCR analysis (SYBR green; Takara). ACTB mRNA expression was utilized as the normalization control. The primers used are listed in Table 1.

**Table 1:** Sequences of primers in qPCR

Gene symbol	Forward (5′-3′)	Reverse (5′-3′)
VEGFR1	TTTGCCTGAAATGGTGAGTAAGG	TGGTTTGCTTGAGCTGTGTTC
VEGFR2	GGCCCAATAATCAGAGTGGCA	CCAGTGTCATTTCCGATCACTTT
NRP1	GGCGCTTTTCGCAACGATAAA	TCGCATTTTTCACTTGGGTGAT
GZMB	CCCTGGGAAAACACTCACACA	GCACAACTCAATGGTACTGTCG
GZMK	GGTGTTCTGATTGATCCACAGT	TGTGCGCCTAAAACCACAGT
PRF1	GTGGGACAATAACAACCCCAT	TGGCATGATAGCGGAATTTTAGG
ACTB	GCCTCGCCTTTGCCGA	CCCACCATCACGCCCTGG

### RNA-seq and analysis

PBMC samples from 20 GO and 20 GH patients were subjected to RNA-seq as described in our previous study [15]. After quality control, the “DESeq2” R package was used to identify differentially expressed genes (DEGs) [16]. DEGs with a fold change ≥ 2 (|log_2_FC|> 1) and a false discovery rate (FDR) < 0.05 were considered significant. A protein–protein interaction (PPI) network was constructed with data from the STRING database (https://string-db.org/) and visualized with Cytoscape (version 3.8.0) [17]. The R package “clusterProfiler” (version 4.0.5) was used to perform gene set enrichment analysis (GSEA) [18]. Gene sets in the Molecular Signatures Database (MSigDB) were selected as the reference gene sets, and a *P* value < 0.01 was set as the threshold [19]. The RNA-seq data reported in this paper have been deposited in the OMIX, China National Center for Bioinformation / Beijing Institute of Genomics, Chinese Academy of Sciences (https://ngdc.cncb.ac.cn/omix: accession no.OMIX002526).

### scRNA-seq data analysis

Three scRNA-seq datasets containing CD4 CTLs, GSE106543, GSE149652, and GSE179292, were obtained from the Gene Expression Omnibus (GEO) database [20–22]. The details for the 3 datasets are listed in Table 2. Our previous scRNA-seq dataset for CD4^+^ T cells from GO patients (SRP226183) combined with the 3 scRNA-seq datasets were integrated for analysis by the “Seurat” R package via the canonical correlation analysis (CCA) method (version 4.0) [13, 23]. The DEGs were ranked by *P* value from smallest to largest. The top 20 significant genes for each cluster were selected for display in a heatmap. The “clusterprofiler” R package was utilized to perform GSEA [18]. Pseudotime trajectory analysis was conducted with the “monocle” R package (version 2.22.0) [24]. Single-sample (ss)GSEA was executed with the R package escape (version 1.4.0), and the results were visualized with the “dittoSeq” R package (version 1.6.0) [25].

**Table 2:** Details of scRNA-seq datasets analyzed

Dataset	Subject	Cell type	References
GSE106543	HC PBMC	T_emra_	[20]
GSE149652	Bladder tumor/normal tissue	CD4^+^ T cells	[21]
GSE179292	CRSwNP nasal polyp tissue	CD4^+^ T cells & ILC2	[22]
SRP226183	GO/GH PBMC	CD4^+^ T cells	[13]

*HC* Healthy controls, *PBMC* Peripheral blood mononuclear cells, *T*_*emra*_ Effector memory T cells re-expressing CD45RA, *CRSwNP* Chronic rhinosinusitis with nasal polyps, *ILC2* Group 2 innate lymphoid cells, *GO* Graves orbitopathy, *GH* Graves hyperthyroidism

### Statistical analysis

Statistical analyses were performed with GraphPad Prism 9.3.1 (GraphPad Software) or R 4.1.2. After a normality test was performed, 2-group data were analyzed using Student’s t test or the Mann‒Whitney test. For multigroup data, one-way ANOVA was utilized. Data are presented as mean ± SEM. A *P* value < 0.05 was considered statistically significant.

## Results

### VEGF-A contributes to the enhanced cytotoxicity of CD4 CTLs in GO

**Fig. 1:**
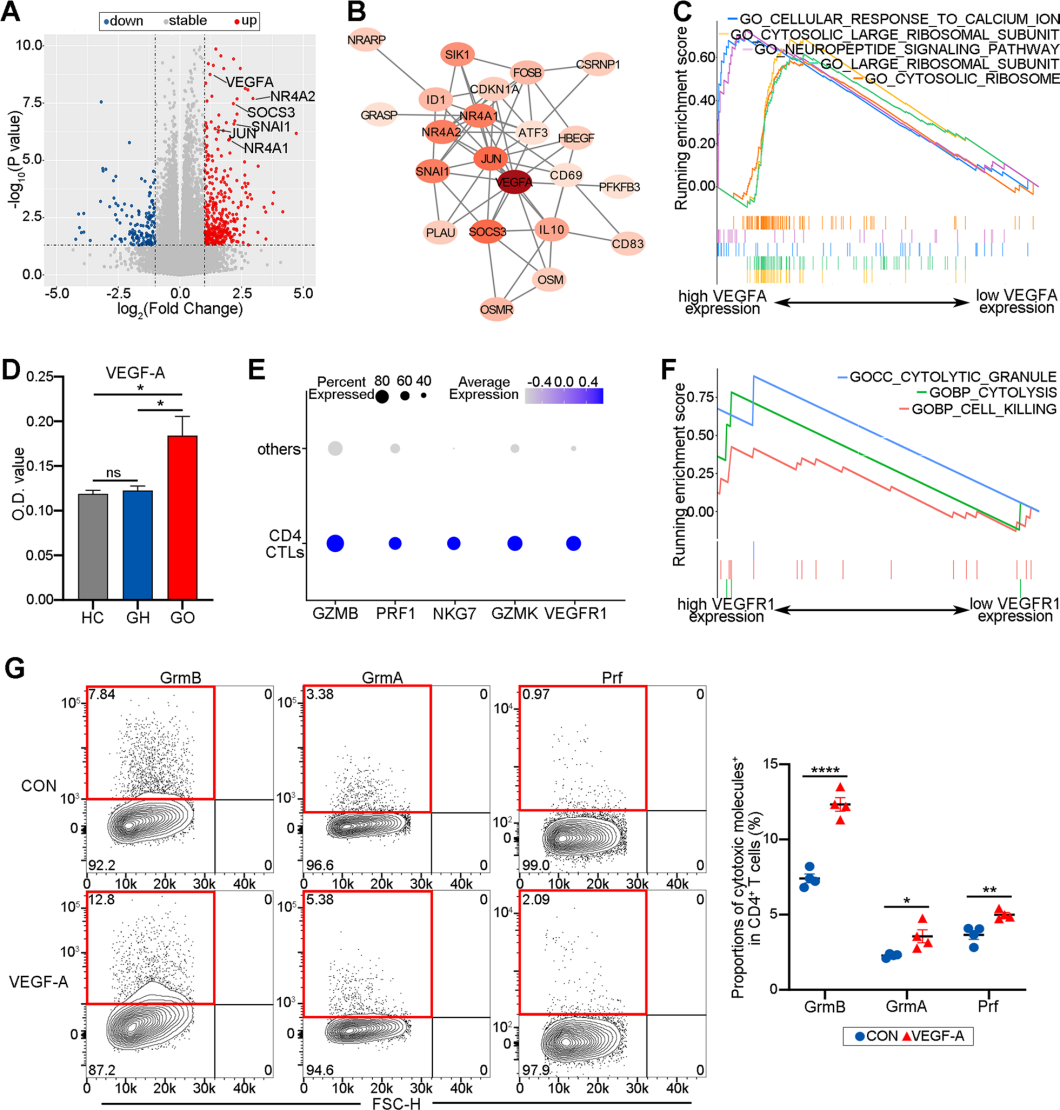
VEGF-A contributes to the enhanced cytotoxicity of CD4 CTLs in GO. **A** Volcano plot showed differentially expressed genes between GO and GH group. Blue dots represented downregulated genes and red dots were upregulated genes. **B** PPI network of potential key genes. The shade of colors denoted the degree of the corresponding gene. **C** Gene set enrichment analysis (GSEA) showed cellular response to calcium ion process was obviously enriched in GO patients with higher expression of VEGFA compared with lower VEGFA expression. **D** Bar plot exhibited the O.D. value of VEGF-A in serum from HC, GH and GO patients (N = 3). **E** Dot plots showed the expression of GZMB, PRF1, NKG7, GZMK and VEGFR1 in CD4 CTLs and non-CD4 CTLs (other) group from GO patients respectively. Color scale represented z-score and dot size represented percentages of cells. **F** GSEA showed cytotoxic related processes were obviously enriched in CD4 CTLs from GO patients with higher expression of VEGFR1 compared with lower VEGFR1 expression. **G** Representative flow cytometry plots showed the ratios of GrmB^+^, GrmA^+^ and Prf^+^ cells in CD4^+^ T cells in the CON and VEGF-A group (N = 4). The red rectangle and number denoted in it meant GrmB^+^, GrmA^+^ and Prf^+^ subsets and specific ratio respectively. The quantification of the proportions of GrmB^+^, GrmA^+^ and Prf^+^ in CD4^+^ T cells was displayed on the right. Blue was CON and red for VEGF-A treated. Error bars showed SEM. The data were representative of at least three biological replicates. HC: healthy control; GH: Graves hyperthyroidism; GO: Graves orbitopathy; CON: control; Grm: granzyme; Prf: perforin. *P < 0.05, **P < 0.01, ****P < 0.0001

In a prior work, we found that GO patients contained CD4 CTLs that expressed more cytotoxic molecules than those from GH patients [13]. To investigate the regulators of the cytotoxicity of CD4 CTLs, transcriptome sequencing was carried out on PBMCs from GO and GH patients. There were 476 upregulated genes and 94 downregulated genes detected (Fig. 1A). After PPI analysis of the DEGs, VEGFA was identified as a potential key gene in GO (Fig. 1B). Furthermore, in the GO patients with higher VEGFA expression, the cellular response to the calcium ion process was upregulated (Fig. 1C). Additionally, VEGF-A was shown to be elevated at the protein level in the plasma of GO patients compared to that of GH patients and HCs (Fig. 1D; *P* = 0.0359 and 0.0284). Considering that the calcium ion response is involved in the synthesis and release of cytotoxic molecules in T cells, we hypothesized that VEGF-A contributed to the increased cytotoxic function of CD4 CTLs in GO [26]. Reanalyzing scRNA-seq data for GO patients was performed to confirm this theory, and it was discovered that GO CD4 CTLs with greater levels of GZMB, PRF1, NKG7, and GZMK expression had upregulated VEGFR1 (Fig. 1E). Furthermore, GSEA results showed that cytotoxic granule, cytolysis, and cell killing processes were enriched in GO CD4 CTLs with upregulated VEGFR1 expression (Fig. 1F). In addition, the proportions of cells expressing cytotoxic molecules such as GrmB, GrmA, and Prf were significantly increased after administrating VEGF-A to CD4^+^ T cells from GO patients (Fig. 1G; *P* < 0.0001, = 0.0286 and 0.0066, respectively). In this way, VEGF-A contributes to the enhanced cytotoxicity of CD4 CTLs in GO and can increase the production of cytotoxic molecules by these cells.

### VEGF-A promotes the cytotoxic function of CD4 CTLs

**Fig. 2:**
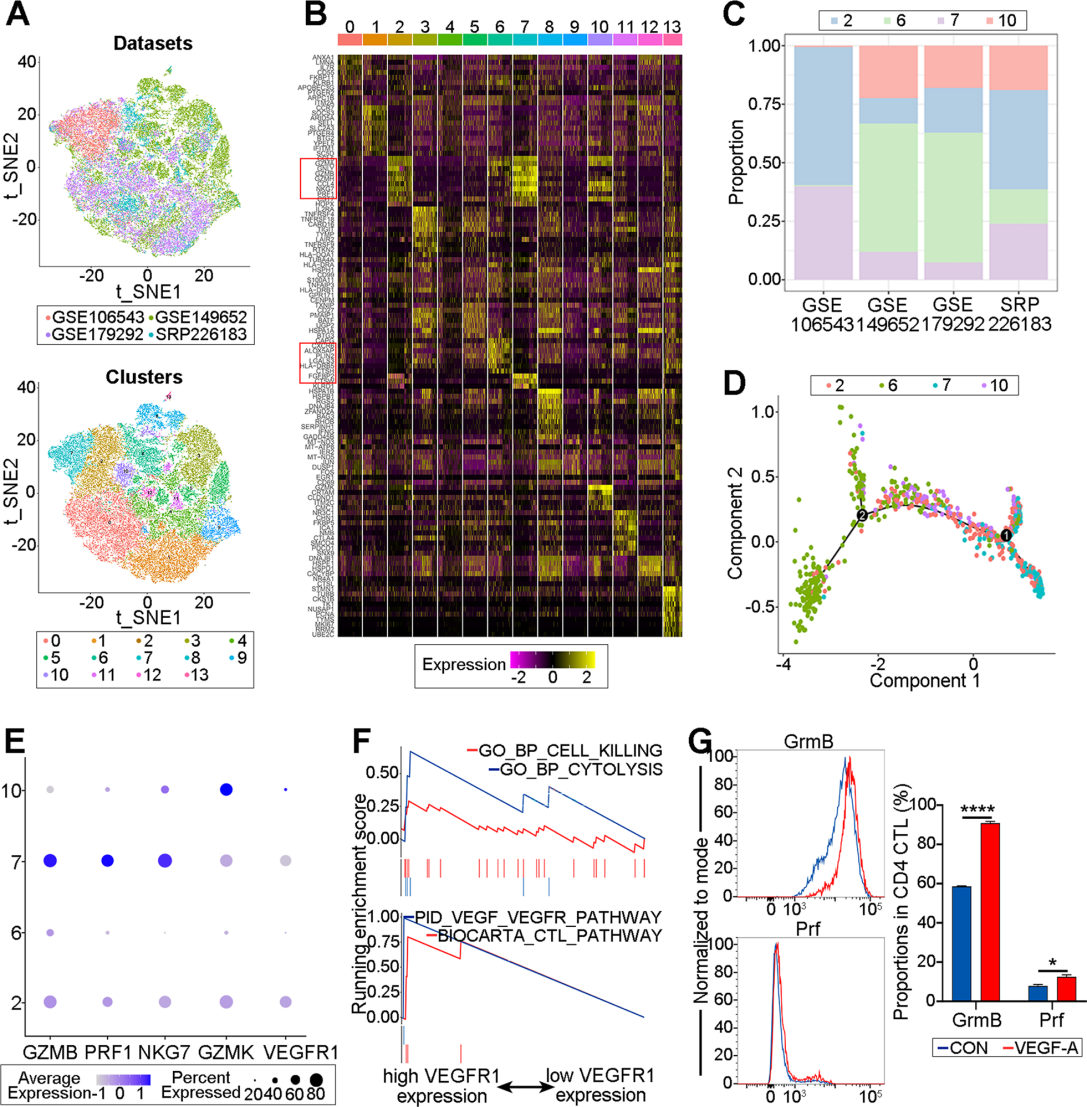
VEGF-A promotes the cytotoxic function of CD4 CTLs. **A** The graph-based clustering and t-SNE algorithm were applied in CD4^+^ T cells from GSE106543 (pink), GSE149652 (green), GSE179292 (purple) and SRP226183 (blue). Clusters denoted by the same color scheme were labeled with inferred cell types (bottom). **B** Heatmap from scRNA-seq analysis via expression data of the top 20 genes differentially expressed for each cluster (denoted by colored bars at the top). Key genes for each cell type were shown on the left margin. Genes in red rectangle represented cytotoxicity related. **C** The stack plots showed the ratio of each cluster of CD4 CTLs. Each color represented different cell cluster. **D** Pseudo-time trajectory analysis showed the differentiation stage of each CD4 CTLs subset according to the cytotoxicity. Pink represented cluster 2, green was cluster 6, blue was cluster 7 and purple was cluster 10. **E** Dot plots showed the expression of GZMB, PRF1, NKG7, GZMK and VEGFR1 in each CD4 CTLs subset respectively. Color scale represented z-score and dot size represented percentages of cells. **F** GSEA showed (upper) cytotoxic related processes and (bottom) VEGF and cytotoxicity pathways were obviously enriched in CD4 CTLs with higher expression of VEGFR1 compared with lower VEGFR1 expression. **G** Representative histogram plots showed the expression of GrmB and Prf in CD4 CTLs in the CON and VEGF-A group. The quantification of the proportions of GrmB^+^ and Prf^+^ in CD4 CTLs was displayed on the right (N = 3). Blue was CON and red for VEGF-A treated. Error bars showed SEM. The data were representative of at least three biological replicates. CON: control; Grm: granzyme; Prf: perforin. *P < 0.05, ****P < 0.0001

Three scRNA-seq datasets containing enriched CD4 CTLs (effector memory T cells re-expressing CD45RA, Temra) or CD4^+^ T cells were downloaded from the GEO database, and integrated analysis was performed with our previous scRNA-seq data (SRP226183) related to GO to further explore the role of VEGF-A in pan CD4 CTLs (Fig. 2A, Table 2). In total, 14 clusters were found (Fig. 2A). Among them, cluster 2, 6, 7, and 10 were recognized as CD4 CTLs due to the upregulated expression of cytotoxic molecules (Fig. 2B). In the HC and GO datasets (GSE106543 and SRP226183), CD4 CTLs with increased cytotoxicity (cluster 2 and 7) predominated, but these cells did not predominate in the bladder cancer or chronic rhinosinusitis with nasal polyps (CRSwNP) datasets (GSE149652 and GSE179292; Fig. 2C&E). It was assumed that CD4 CTLs in lesions tended to be exhausted with decreased cytotoxicity given that pseudotime trajectory analysis suggested that cluster 6 and 10 might differ from cluster 2 and 7 (Fig. 2D; Additional file 1: Fig. S1). Notably, CD4 CTLs with greater cytotoxicity had upregulated VEGFR1 (clusters 2 and 7; Fig. 2E). Through GSEA, it was discovered that cluster 2 and 7, which had higher VEGFR1 expression, exhibited upregulation of processes related to VEGF_VEGFR signaling and cytotoxicity (Fig. 2F). Furthermore, in contrast to cells treated with the blank control, VEGF-A-treated CD4 CTLs from HCs produced more GrmB and Prf (CON; Fig. 2G; *P* < 0.0001 and *P* = 0.0211). These findings demonstrate that VEGF-A is involved in the cytotoxicity of CD4 CTLs and promotes their cytotoxic function.

### VEGF-R1/R2 correlates with the cytotoxic function of CD4 CTLs

**Fig. 3:**
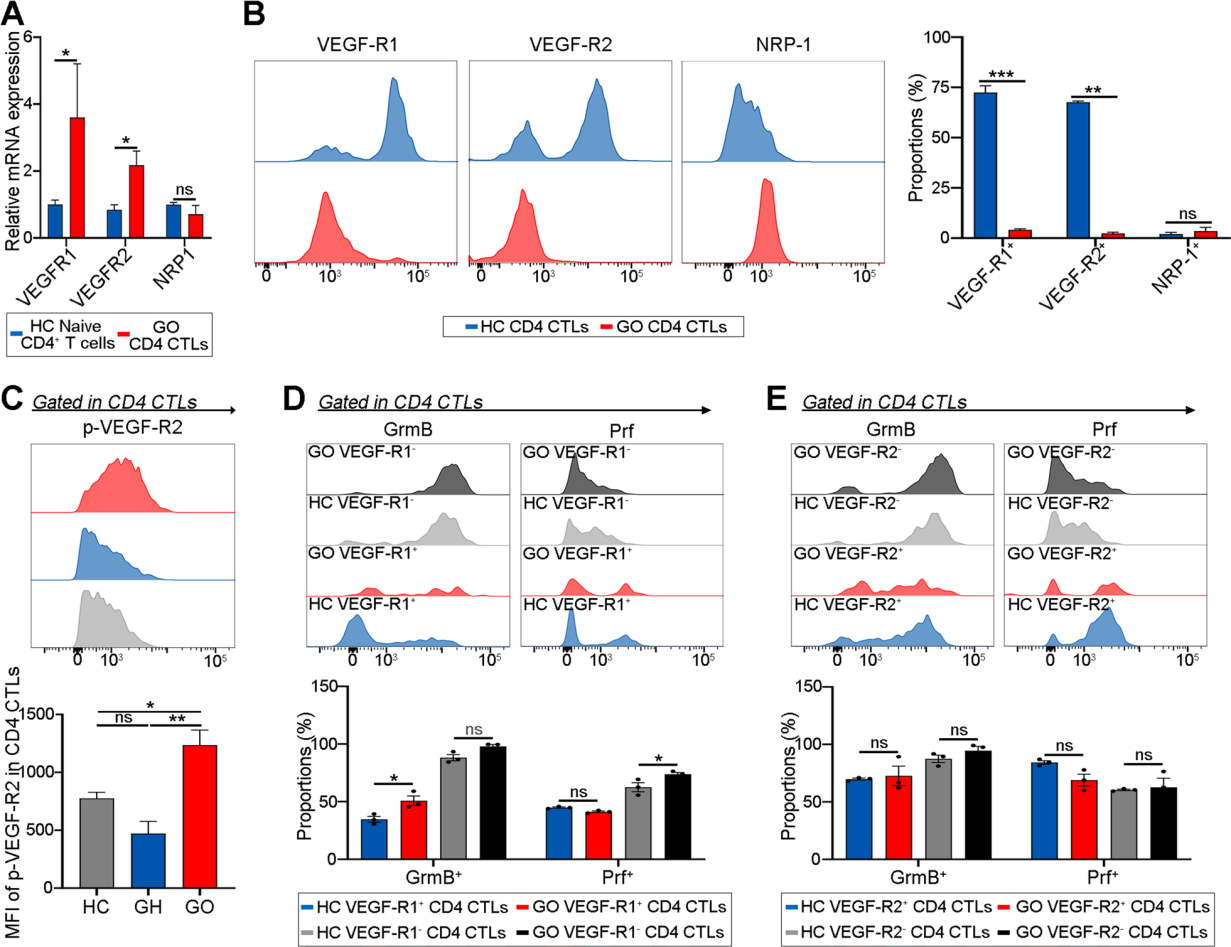
VEGF-R1/R2 correlates with the cytotoxic function of CD4 CTLs. **A** Bar plots exhibited levels of VEGFR1, VEGFR2 and NRP1 in CD4 CTLs (red) from GO patients and CD4^+^ Naive T cells (blue) from HC (N = 4). **B** Representative histogram plots exhibited the expression of VEGF-R1, VEGF-R2 and NRP-1 in CD4 CTLs from GO (red) and HC (blue) (N = 3). The bar plots showed the proportions of VEGF-R1^+^, VEGF-R2^+^ and NRP-1^+^ cells in CD4 CTLs from GO (red) and HC (blue). **C** Representative histogram plots (upper) exhibited the expression of p-VEGF-R2 in CD4 CTLs from GO (red), GH (blue) and HC (gray) (N = 3). The bar plots (bottom) showed the MFI of p-VEGF-R2 in CD4 CTLs from GO (red), GH (blue) and HC (gray) group. **D**, **E** Representative histogram plots (upper) exhibited the expression of GrmB and Prf in (**D**) VEGF-R1/ (**E**) VEGF-R2 ^±^CD4 CTLs from GO (red and black) and HC (blue and gray) patients. The bar plots (bottom) showed the proportions of GrmB^+^ and Prf^+^ cells in (**D**) VEGF-R1/(**E**) VEGF-R2 ^±^CD4 CTLs from GO and HC group (N = 3). Blue denoted HC VEGF-R1/2^+^ CD4 CTLs, red was GO VEGF-R1/2^+^ CD4 CTLs, gray was HC VEGF-R1/2^−^ CD4 CTLs and black was GO VEGF-R1/2^−^ CD4 CTLs. Error bars showed SEM. The data were representative of at least three biological replicates. HC: healthy control; GH: Graves hyperthyroidism; GO: Graves orbitopathy; Grm: granzyme; Prf: perforin; p-VEGF-R2: phosphorylated VEGF-R2. *P < 0.05, **P < 0.01, ***P < 0.001

Although preliminary evidence suggests that VEGF-A regulates CD4 CTLs, the specific VEGF-R involved remains unknown. To clarify the downstream receptors, qPCR was performed to measure the expression of well-known VEGF-A receptors including VEGFR1, VEGFR2, and NRP1 on CD4 CTLs with increased cytotoxicity. Because GO CD4^+^ T cells expressed much more cytotoxic molecules than those from HCs, GO CD4 CTLs were used as an example of CD4 CTLs with increased cytotoxicity (Additional file 2: Fig. S2; GrmB: *P* = 0.0083; Prf: *P* = 0.0301). The results revealed that VEGFR1 and VEGFR2 were upregulated in GO CD4 CTLs with increased cytotoxicity compared with HC naïve CD4^+^ T cells (Fig. 3A; *P* = 0.0286, 0.0409 and 0.4857, respectively). In contrast, there were lower proportions of VEGF-R1^+^ and VEGF-R2^+^ cells in GO CD4 CTLs with higher cytotoxicity than in HC CD4 CTLs, as measured by flow cytometry (Fig. 3B; VEGF-R1: *P* = 0.0001; VEGF-R2: *P* = 0.0016; NRP-1: *P* = 0.9240). We hypothesized that the decrease in the level of VEGF-R1/R2 expression on GO CD4 CTLs with increased cytotoxicity was caused by binding with overexpressed VEGF-A because VEGF-R1/R2 levels were reported to be diminished following binding to VEGF [27]. As expected, GO CD4 CTLs with higher cytotoxicity had higher levels of phosphorylated (p)-VEGF-R2 than CD4 CTLs from HCs or GH patients (Fig. 3C; *P* = 0.0480 and 0.0041), indicating that VEGF/VEGF-R2 signaling was upregulated in CD4 CTLs with enhanced cytotoxicity.

Furthermore, the relationships between the expression of VEGF-R1/R2 and cytotoxic molecules were examined. When VEGF-R1^+^ GO CD4 CTLs with higher cytotoxicity were compared to VEGF-R1^+^ HC CD4 CTLs, it was revealed that the proportions of GrmB^+^ cells were increased, while no differences were detected between GO and HC CD4 CTLs with VEGF-R1^−^ or VEGF-R2^±^ (Fig. 3D&E; *P* = 0.0184, 0.1594, 0.9745 and 0.7348). With respect to the proportion of Prf^+^ cells, there were no differences between VEGF-R1^+^ or VEGF-R2^±^ GO and HC CD4 CTLs (Fig. 3D&E; *P* = 0.6380, 0.1858, and 0.9856). Interestingly, VEGF-R1^−^ GO CD4 CTLs had higher Prf expression than VEGF-R1^−^ HC CD4 CTLs (Fig. 3D; *P* = 0.0230). Hence, VEGF-R1/R2 signaling is upregulated in CD4 CTLs with increased cytotoxicity and correlated with higher cytotoxicity to some extent.

### VEGF-A enhances the cytotoxic function of CD4 CTLs *via* VEGF-R1/R2

**Fig. 4:**
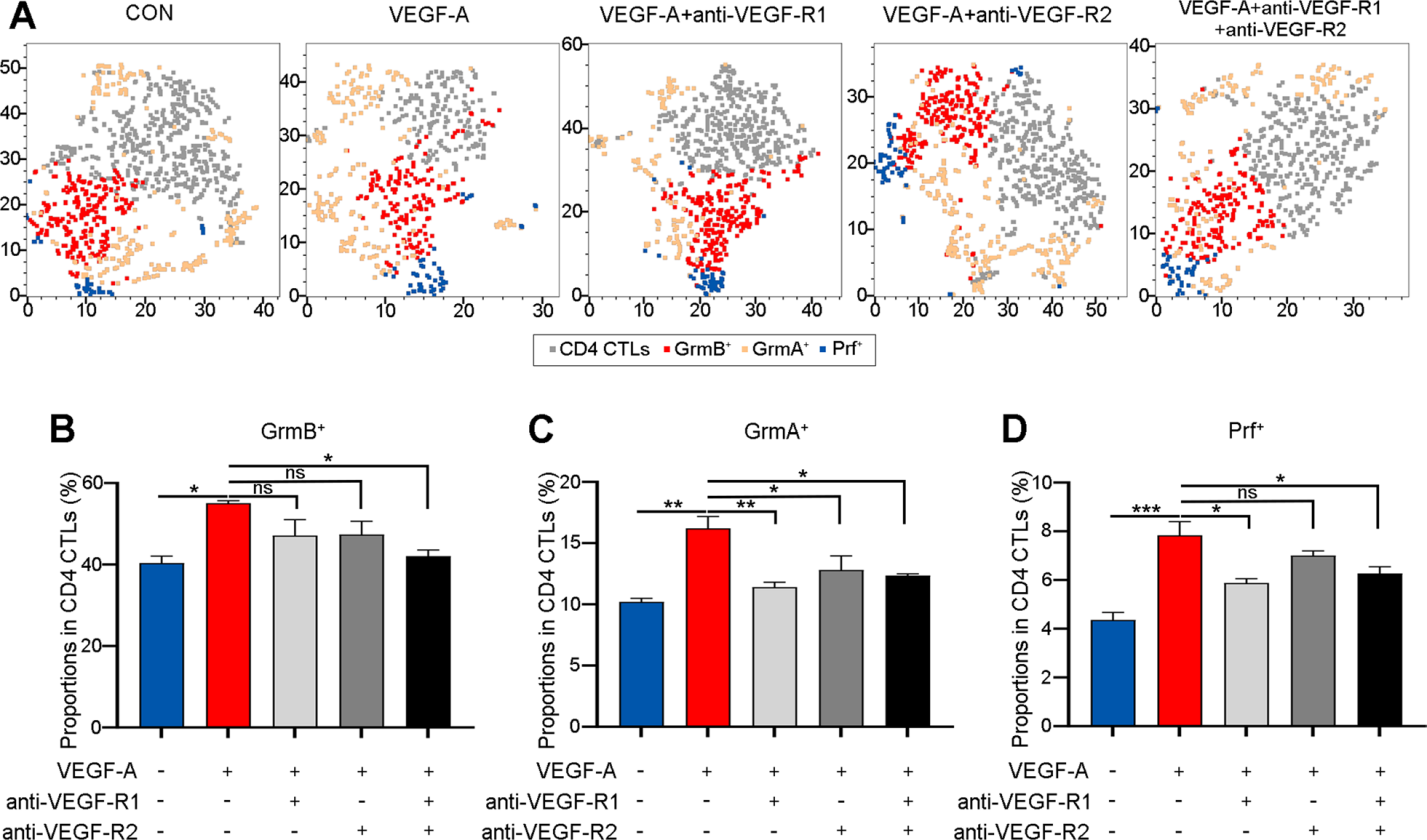
VEGF-A enhances the cytotoxic function of CD4 CTLs via VEGF-R1/R2. **A** The representative tSNE plots showed the proportions of GrmB^+^ (red), GrmA^+^ (yellow) and Prf^+^ (blue) in CD4 CTLs (gray). **B–D** The quantification of the ratio of **B** GrmB^+^, **C** GrmA^+^ and **D** Prf^+^ cells in CD4 CTLs was shown in the bar plots (N = 3). Blue was CON, red for VEGF-A treated, light gray for VEGF-A + anti-VEGF-R1 neutralizing antibody, dark gray for VEGF-A + anti-VEGF-R2 neutralizing antibody and black for VEGF-A + anti-VEGF-R1 + anti-VEGF-R2 neutralizing antibody group. Error bars showed SEM. The data were representative of at least three biological replicates. CON: control; Grm: granzyme; Prf: perforin. *P < 0.05, **P < 0.01, ***P < 0.001

To identify the functional downstream receptors of VEGF-A in CD4 CTLs with increased cytotoxicity, CD4^+^ T cells from GO patients were treated with anti-VEGF-R1/R2 neutralizing antibodies. After VEGF-R1 was blocked, the enhancement of GrmA and Prf production in CD4 CTLs induced by VEGF-A was suppressed (Fig. 4A, C, D; *P* = 0.0055 and 0.0128). Furthermore, only the proportion of GrmA^+^ cells in CD4 CTLs was diminished in the VEGF-A + anti-VEGF-R2 group compared to the VEGF-A group (Fig. 4A, C; *P* = 0.0450). Additionally, the expression of GrmB, GrmA and Prf in CD4 CTLs was significantly decreased in the group with VEGF-R1 and VEGF-R2 blocked simultaneously in comparison to the VEGF-A group (Fig. 4A–D; *P* = 0.0283, 0.0226 and 0.0468, respectively). These findings suggest that VEGF-A concurrently activates VEGF-R1 and VEGF-R2 to induce the production of cytotoxic molecules in CD4 CTLs.

### AKT/mTOR signaling is upregulated in CD4 CTLs with increased cytotoxicity

**Fig. 5:**
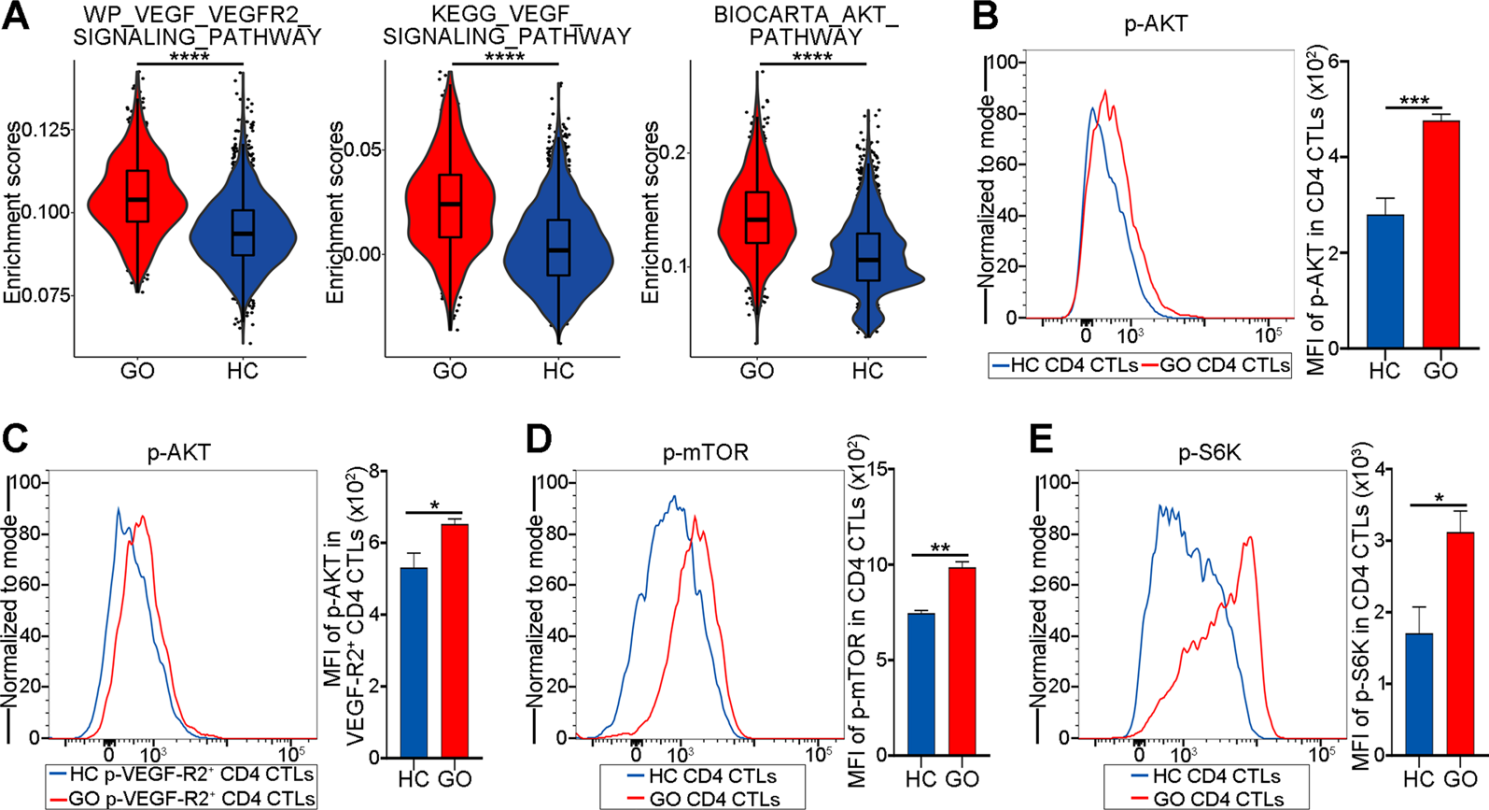
AKT/mTOR signaling is up-regulated in CD4 CTLs with increased cytotoxicity. **A** Violin plots exhibited ssGSEA enrichment scores of VEGF signaling and AKT related pathways in CD4 CTLs from GO patients (red) and HC (blue). **B** Representative histogram plots (left) exhibited the expression of p-AKT in CD4 CTLs from GO (red) and HC (blue) (N = 5). The bar plots (right) showed the MFI of p-AKT in CD4 CTLs from GO (red) and HC (blue). **C** Representative histogram plots (left) exhibited the expression of p-AKT in p-VEGF-R2^+^ CD4 CTLs from GO (red) and HC (blue) (N = 4). The bar plots (right) showed the MFI of p-AKT in p-VEGF-R2.^+^ CD4 CTLs from GO (red) and HC (blue). **D**, **E** Representative histogram plots exhibited the expression of **D** p-mTOR and **E** p-S6K in CD4 CTLs from GO (red) and HC (blue) (N = 3). The bar plots showed the MFI of **D** p-mTOR and **E** p-S6K in CD4 CTLs from GO (red) and HC (blue). Error bars showed SEM. The data were representative of at least three biological replicates. ssGSEA: single sample gene score enrichment analysis; HC: healthy control; GO: Graves orbitopathy; mTOR: mammalian target of rapamycin; S6K: ribosomal protein S6 kinase. *P < 0.05, **P < 0.01, ***P < 0.001, ****P < 0.0001

The scRNA-seq data was used to further investigate downstream signaling through ssGSEA of the enriched pathways between CD4 CTLs with higher and lower cytotoxicity. In CD4 CTLs with enhanced cytotoxicity, the AKT pathway was upregulated in addition to the VEGF signaling pathway (Fig. 5A; *P* < 0.0001). AKT signaling might be involved in the impact of VEGF-A-VEGF-R2 on CD4 CTLs with increased cytotoxicity, as evidenced by the MFI of p-AKT being considerably increased in GO CD4 CTLs with increased cytotoxicity, particularly in p-VEGF-R2^+^ GO CD4 CTLs (Fig. 5B-C; *P* = 0.0006 and 0.0290). In addition, the MFIs of p-mTOR and p-S6K were obviously elevated in CD4 CTLs with enhanced cytotoxicity (Fig. 5D–E; P = 0.0016 and 0.0394), which indicated the activation of mTOR signaling. Therefore, the AKT/mTOR pathway may contribute to the effects of VEGF-A on CD4 CTLs with elevated cytotoxicity.

### *VEGF-A promotes the cytotoxic function of CD4 CTLs *via* the AKT/mTOR pathway*

**Fig. 6:**
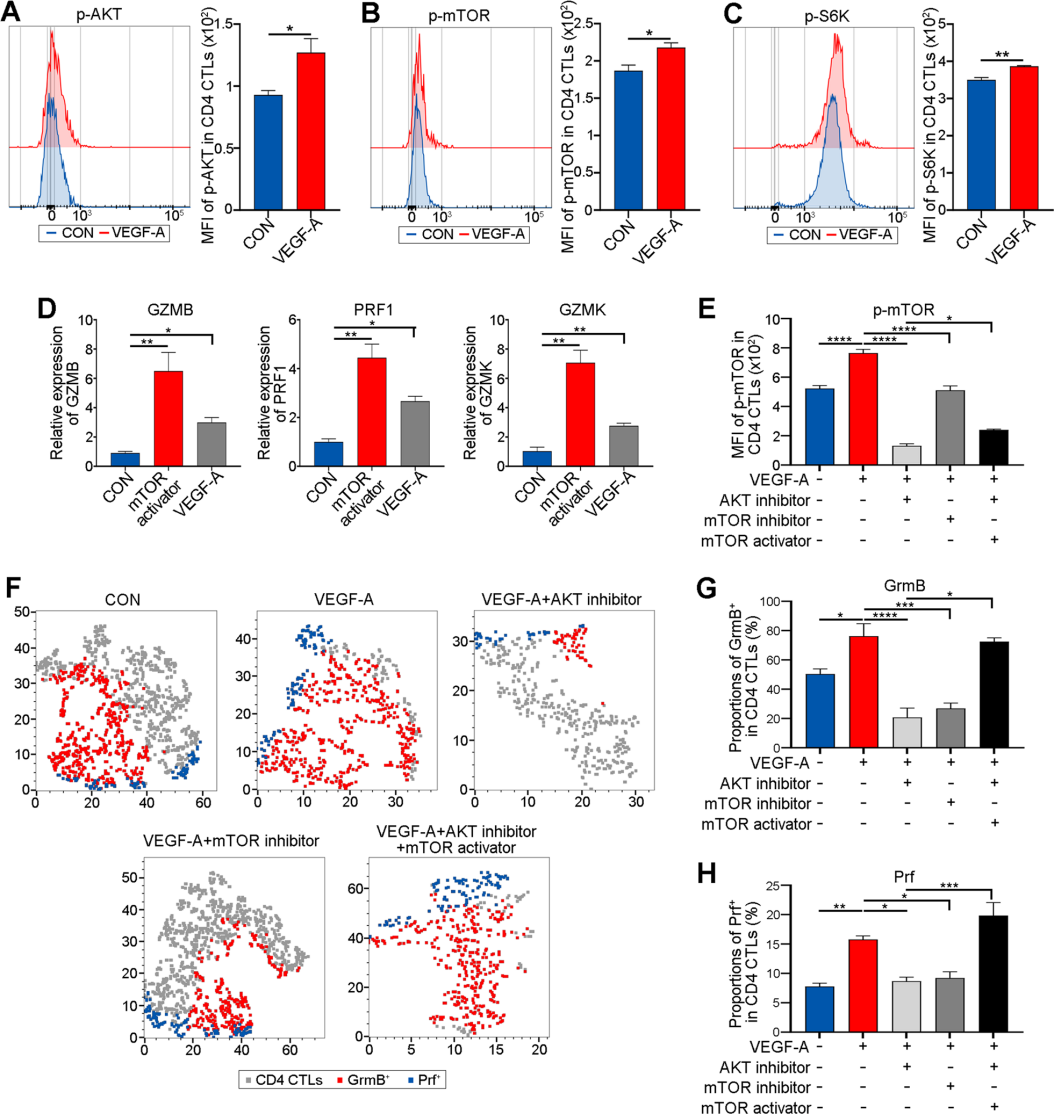
VEGF-A promotes the cytotoxic function of CD4 CTLs via AKT/mTOR pathway. **A**, **B** Representative histogram plots (upper) exhibited the expression of **A** p-AKT **B** p-mTOR and **C** p-S6K in CD4 CTLs from CON (blue) and VEGF-A (red) group (N = 3). The bar plots (bottom) showed the MFI of **A** p-AKT **B** p-mTOR and **C** p-S6K in CD4 CTLs from CON (blue) and VEGF-A (red) group. **D** Bar plots exhibited levels of GZMB, PRF1 and GZMK in CD4 CTLs from CON (blue), mTOR activator (red) and VEGF-A (gray) group (N = 4). **E–H** The levels of **E** p-mTOR and **F–H** the proportions of GrmB^+^ and Prf^+^ in CD4 CTLs (N = 4). **F** The representative tSNE plots showed the proportions of GrmB^+^ (red) and Prf^+^ (blue) in CD4 CTLs (gray). The quantification of **E** the MFI of p-mTOR and ratio of **G** GrmB^+^ and **H** Prf.^+^ cells in CD4 CTLs was shown in the bar plots. Blue was CON, red for VEGF-A treated, light gray was VEGF-A + AKT inhibitor, dark gray for VEGF-A + mTOR inhibitor, and black was VEGF-A + AKT inhibitor + mTOR activator group. Error bars showed SEM. The data were representative of at least three biological replicates. CON: control; mTOR: mammalian target of rapamycin; Grm: granzyme; Prf: perforin. *P < 0.05, **P < 0.01, ***P < 0.001, ****P < 0.0001

To determine whether the impacts of VEGF-A on CD4 CTLs were the cause of the activated AKT/mTOR signaling observed, the expression of p-AKT, p-mTOR and p-S6K was examined in CD4 CTLs treated with the blank control (CON) or VEGF-A. As anticipated, the MFIs of p-AKT, p-mTOR, and p-S6K were dramatically increased in the VEGF-A-treated CD4 CTLs (Fig. 6A; *P* = 0.0460, *P* = 0.0344 and 0.0047). In addition, at the gene expression level, there were obvious enhancements of GZMB, PRF1, and GZMK in the mTOR activator group compared with the CON group (Fig. 6D; *P* = 0.0048, 0.0010 and 0.0004 respectively). Moreover, after treating CD4 CTLs with an AKT inhibitor (MK-2206 2HCl) or mTOR inhibitor (rapamycin), the increases in the MFI of p-mTOR as well as the proportions of GrmB^+^ and Prf^+^ cells in CD4 CTLs induced by VEGF-A were significantly diminished, indicating that VEGF-A activates mTOR signaling via AKT and the facilitating effects of VEGF-A on the cytotoxicity of CD4 CTLs were achieved by AKT and mTOR signaling (Fig. 6E–H; AKT inhibitor: *P* < 0.0001, < 0.0001 and = 0.0137; mTOR inhibitor: *P* < 0.0001, = 0.0002 and = 0.0140). Meanwhile, when an mTOR activator (MHY1485) was administered, the decrease in the MFI of p-mTOR as well as the proportions of GrmB^+^ and Prf^+^ cells in VEGF-A treated CD4 CTLs caused by the suppressed AKT could be rescued, implying the crucial role of mTOR in the downstream of AKT pathway in CD4 CTLs (Fig. 6E–H; P = 0.0296, < 0.0001 and = 0.0003). These results suggest that VEGF-A can enhance the cytotoxicity of CD4 CTLs via the AKT/mTOR pathway.

## Discussion

In the current study, we identified that VEGF-A could induce the expression of cytotoxic molecules in CD4 CTLs via its receptors VEGF-R1/2 and this effect was achieved via the AKT/mTOR signaling pathway. These data reveal that VEGF-A is a regulator of the cytotoxicity of CD4 CTLs and provide insights for the development of novel treatments for disorders associated with CD4 CTLs.

VEGF-A, which is produced by the majority of cells, is a crucial member of the VEGF cytokine family, along with VEGF-B, VEGF-C, VEGF-D and placental growth factor (PIGF) [14]. Although its canonical biological function is angiogenesis mediated by regulating endothelial growth and vascular permeability, VEGF-A is also a mediator of T-cell function. When present in tumor-bearing individuals, VEGF-A has an immunosuppressive function that can inhibit the antitumor activity of CD8^+^ T cells within tumors by aggravating the hypoxic microenvironment, activating an exhaustion-specific transcription program and prompting the proliferation and activity of regulatory T cells [28–30]. Furthermore, various effects of VEGF-A on T cells have been identified by several researchers. In combination with a cognate antigen, VEGF-A has been shown to trigger proinflammatory responses, including Th1, Th2 and Th17 activities, in vitro and/or in vivo (an asthma mouse model) [31–33]. Additionally, improved T-cell adhesion was observed in inflammatory bowel disease when high-dose VEGF-A was administered, which subsequently accelerated disease progression [34]. Notably, consistent with our findings that VEGF-A could enhance the cytotoxic function of CD4 CTLs (the majority of which exhibit a memory phenotype), VEGF has been shown to boost the immune responses of CD4^+^CD45RO^+^ memory T cells by upregulating the phosphorylation and activation of extracellular signal-regulated kinase (ERK) and AKT [35]. Hence, VEGF-A is a pluripotent regulator that can enhance the cytotoxic function of CD4 CTLs.

Regarding VEGF-Rs, there are three major VEGF-A receptors: VEGF-R1, VEGF-R2 and NRP-1. Canonical VEGF signaling is transduced through VEGF-R1/R2, with VEGF-R2 being regarded as the dominant receptor, while NRP-1 is involved in increasing the binding affinity of VEGF-A for VEGF-R2 [14]. Previous studies have revealed that CD4^+^ and CD8^+^ T cells express VEGF-R1 and VEGF-R2 and that this expression increases significantly after T cells are activated [36]. The effects of VEGF-A on the aforementioned T cells are decreased after VEGF-R1/R2 are blocked, which is consistent with our results showing that both VEGF-R1 and VEGF-R2 contribute to VEGF-A signaling [37]. In addition, it was discovered that VEGF-R2 participates in the impacts of VEGF-A on natural killer (NK) cells, enhancing cytotoxic activity, which agrees with our results showing that VEGF-A enhances the cytotoxicity of CD4 CTLs via VEGF-R2 [38]. However, in this study, neutralizing antibodies were applied to block VEGF-Rs, and further investigation utilizing gene editing techniques in vitro and in vivo is needed. Additionally, the expression of VEGF-R1/R2 on the surface of CD4 CTLs with increased cytotoxicity was found to be reduced, while that of p-VEGF-R2 increased obviously, which implied the existence of ligand-activated endocytosis of VEGF-R2, as previously reported [27].

Given that AKT/mTOR signaling is a potential downstream cascade of VEGF stimulation, we identified the activation of the AKT/mTOR/S6K pathway in higher-cytotoxicity and VEGF-A-treated CD4 CTLs [39]. Application of the mTOR inhibitor rapamycin produced a reduction in the cytotoxicity of CD4 CTLs caused by VEGF-A, indicating that VEGF-A enhanced the cytotoxic function of CD4 CTLs via the mTOR pathway, which along with our findings that rapamycin could improve GO and suppress CD4 CTLs [40]. It has been reported that the antitumor immunity of CD8^+^ T cells with defective AKT signaling diminished during the memory phase, and AKT cooperates with TCR- and IL-2- signaling to induce transcriptional processes controlling the expression of cytotoxic molecules in CTLs [41, 42]. Similarly, proteomic data for CTLs showed that mTORC1 preferentially repressed or promoted the expression of approximately 10% of CTL proteins [43]. Additionally, mTOR activation in CD8^+^ T cells from rheumatoid arthritis patients was identified, and it was positively linked with the severity of the condition [44]. Therefore, AKT/mTOR signaling is involved in not only the cytotoxicity of CD8 CTLs but also that of CD4 CTLs.

## Conclusions

In conclusion, VEGF-A promotes the cytotoxicity of CD4 CTLs via VEGF-R1/2, and this regulation is achieved by AKT/mTOR signaling. These data reveal that VEGF-A is a mediator of the cytotoxic function of CD4 CTLs and facilitate the development of novel treatments for disorders associated with CD4 CTLs.

## Supplementary Information

**Additional file 1: Fig. S1.** The exhaustion signature was up-regulated in CD4 CTLs with lower cytotoxicity. A GSEA showed PD_1_SIGNALING was obviously enriched in CD4 CTLs with lower cytotoxicity compared with those with higher cytotoxicity (*P* = 0.02192). **B** Dot plots showed the expression of GZMB, PRF1, GZMA, PDCD1, LAG3, TIGIT and CTLA4 in CD4 CTLs with lower cytotoxicity and higher cytotoxicity respectively. Color scale represented z-score and dot size represented percentages of cells.** C** Pseudo-time trajectory analysis showed the expression of PDCD1, LAG3, TIGIT and CTLA4 in different differentiation stage of CD4 CTLs. Color scale represented log10(value + 0.1).

**Additional file 2: Fig. S2.** GO CD4^+^ T cells had more cytotoxic molecules than those from HC. **A-B** Representative flow cytometry plots showed the ratios of **A** GrmB^+^ and **B** Prf^+^ cells in CD4^+^ T cells in the GO and HC group (N = 3). The number denoted in it meant the specific ratio of GrmB^+^ and Prf^+^ subsets. The quantification of the proportions of **A** GrmB^+^ and **B** Prf^+^ in CD4^+^ T cells was displayed in the bar plots. Blue was HC and red for GO. Error bars showed SEM. HC: healthy control; GO: Graves orbitopathy; Grm: granzyme; Prf: perforin. *P < 0.05, **P < 0.01.

## Data Availability

Data supporting the findings of this study are available from the corresponding authors on reasonable request. The RNA-seq data have been deposited in the OMIX, China National Center for Bioinformation / Beijing Institute of Genomics, Chinese Academy of Sciences (https://ngdc.cncb.ac.cn/omix: Accession No.OMIX002526).

## Abbreviations

CD4 CTLs: CD4^+^ cytotoxic T cells
MHC: Major histocompatibility complex
PBMCs: Peripheral blood mononuclear cells
TCR: T cell receptor
ThPok: T-helper-inducing POZ/Krueppel-like factor
Eomes: Eomesodermin
Grm: granzyme
IFN: Interferon
JAK: Janus kinase
STAT: Signal transducers and activators of transcription
GO: Graves orbitopathy
RNA-seq: RNA-sequencing
GH: Graves hyperthyroidism
VEGF: Vascular endothelial growth factor
scRNA-seq: Single-cell RNA sequencing
VEGF-R: VEGF-receptor
HCs: Healthy controls
CON: Control
KLRG1: Killer cell lectin-like G
Prf: Perforin
NRP: Neuropilin
p-: Phosphorylated
S6K: S6 kinase
DEGs: Differentially expressed genes
FC: Fold change
PPI: Protein‒protein interaction
GSEA: Gene set enrichment analysis
GEO: Gene Expression Omnibus
CCA: Canonical correlation analysis
T_emra_: Effector memory T cells re-expressing CD45RA
CRSwRP: Chronic rhinosinusitis with nasal polyps
