# Supplementary figures and images for: VEGF-A enhances the cytotoxic function of CD4+ cytotoxic T cells via the VEGF-receptor 1/VEGF-receptor 2/AKT/mTOR pathway
Source: J Transl Med. 2023 Feb 3;21:74. doi: 10.1186/s12967-023-03926-w (PMC9896805; doi:10.1186/s12967-023-03926-w)

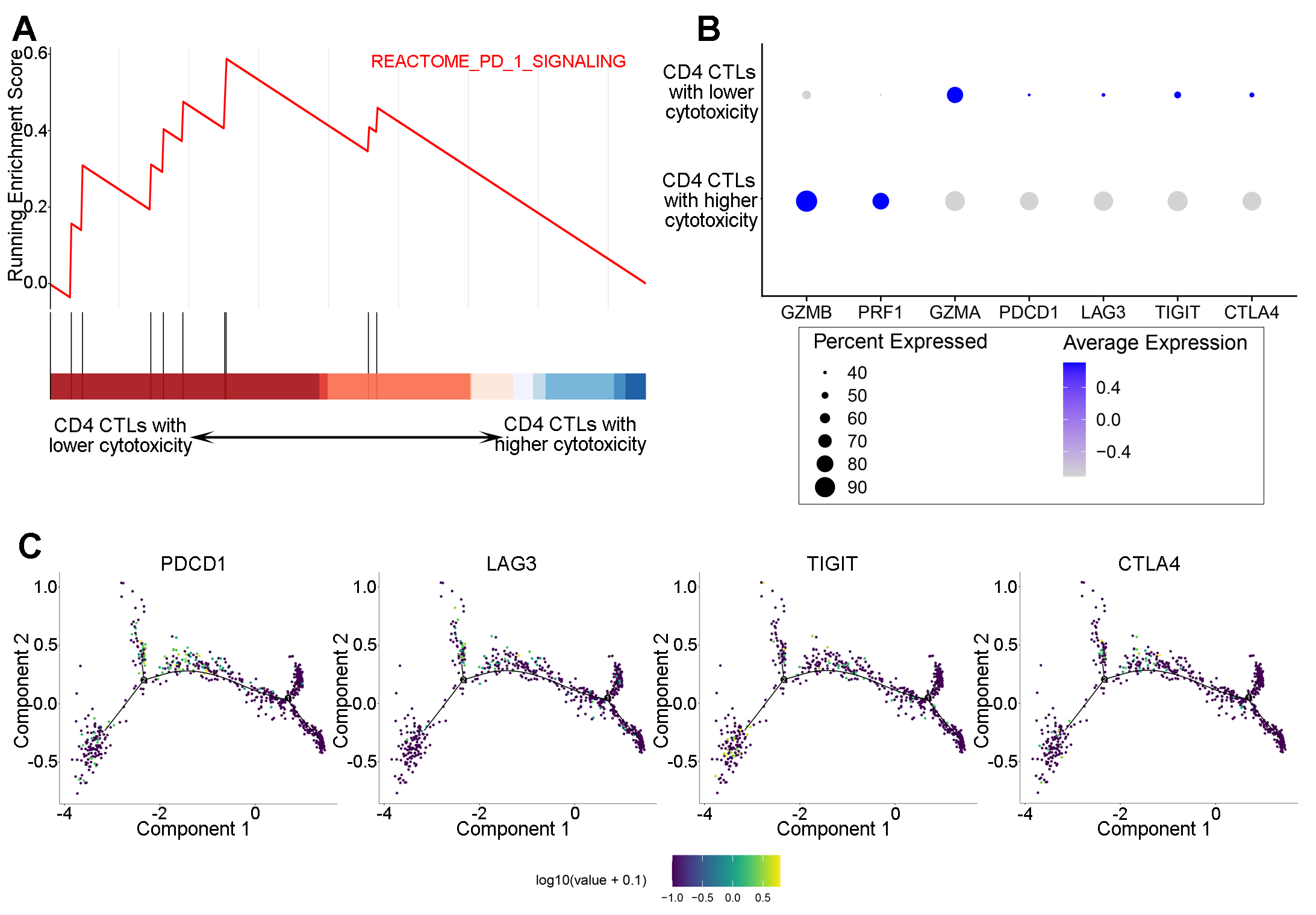

Supplement: Supplementary file 1 — Additional file 1: Fig. S1. The exhaustion signature was up-regulated in CD4 CTLs with lower cytotoxicity. A GSEA showed PD_1_SIGNALING was obviously enriched in CD4 CTLs with lower cytotoxicity compared with those with higher cytotoxicity (P = 0.02192). B Dot plots showed the expression of GZMB, PRF1, GZMA, PDCD1, LAG3, TIGIT and CTLA4 in CD4 CTLs with lower cytotoxicity and higher cytotoxicity respectively. Color scale represented z-score and dot size represented percentages of cells. C Pseudo-time trajectory analysis showed the expression of PDCD1, LAG3, TIGIT and CTLA4 in different differentiation stage of CD4 CTLs. Color scale represented log10(value + 0.1). [file 12967_2023_3926_MOESM1_ESM.tif]

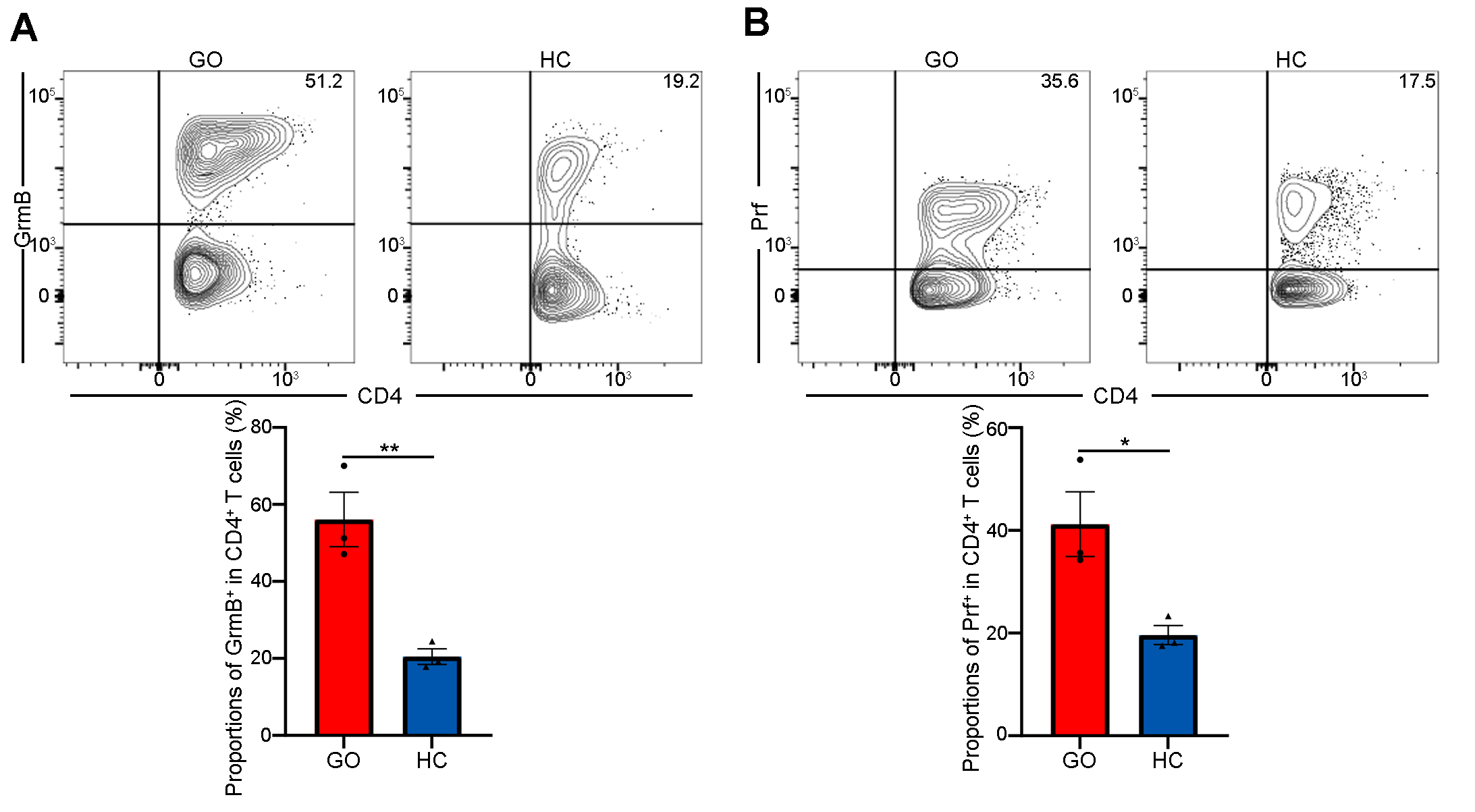

Supplement: Supplementary file 2 — Additional file 2: Fig. S2. GO CD4+ T cells had more cytotoxic molecules than those from HC. A-B Representative flow cytometry plots showed the ratios of A GrmB+ and B Prf+ cells in CD4+ T cells in the GO and HC group (N = 3). The number denoted in it meant the specific ratio of GrmB+ and Prf+ subsets. The quantification of the proportions of A GrmB+ and B Prf+ in CD4+ T cells was displayed in the bar plots. Blue was HC and red for GO. Error bars showed SEM. HC: healthy control; GO: Graves orbitopathy; Grm: granzyme; Prf: perforin. *P < 0.05, **P < 0.01. [file 12967_2023_3926_MOESM2_ESM.tif]
